# The health status of the early care and education workforce in the USA: a scoping review of the evidence and current practice

**DOI:** 10.1186/s40985-019-0117-z

**Published:** 2020-01-08

**Authors:** Laura M. Lessard, Katilyn Wilkins, Jessica Rose-Malm, M. Chaplin Mazzocchi

**Affiliations:** 1 University of Delaware, College of Health Sciences, Newark, DE 19716 USA; 2 Child Care Aware of America, 1515 N. Courthouse Rd—3rd Floor, Arlington, VA 22201 USA

**Keywords:** Child care, Worker health, Health promotion

## Abstract

**Background:**

More than two million early care and education (ECE) providers care for young children in the USA each day. These providers tend to earn low wages and many are enrolled in public assistance programs. Nearly all ECE providers are female and they are disproportionately women of color. Despite the fact that these attributes place the ECE workforce at greater risk of chronic disease, the health status of the workforce is not established and the availability and effectiveness of interventions to improve their health status is also not known.

**Methods:**

We conducted a scoping review of both the published literature and current practice to identify all articles and interventions targeting the health status of the ECE workforce. Our search strategy identified scientific articles published in English within the past 10 years as well as any interventions targeting the ECE workforce that have been implemented within the past 3 years. Data from both scientific articles and practice were extracted using systematic methods and summarized.

**Results:**

Thirteen studies described some component of physical health including diet quality (11 studies), physical activity (8 studies), and height/weight/body mass index (7 studies), and 21 studies assessed component(s) of mental health including depression (15 studies), stress (8 studies), and mindfulness (3 studies). ECE providers reported a high prevalence of overweight, obesity, and chronic disease diagnoses and spend significant time being sedentary, and some report low diet quality. Mental health concerns in this population include depression and high stress. Eleven interventions targeting ECE workforce wellness were also identified; most focused on nutrition, physical activity and/or stress.

**Conclusion:**

The limited evidence available for review describes a workforce in need of health promotion interventions to address high levels of mental and physical health challenges, some above and beyond peers with comparable demographic characteristics. Several promising interventions were identified from both the published and unpublished literature; these interventions should be further implemented and evaluated to assess their impact on the workforce.

## Background

In the United States (USA), more than two million early care and education (ECE) providers care for approximately ten million young children every day [1]. The ECE system in the USA serves children from birth through 6 years of age and includes several program types: child care centers that can be for-profit or non-profit, Early Head Start and Head Start Programs that are funded through grants from the federal government to serve children from low-income households, pre-kindergarten programs available in some jurisdictions for children age 3–4 years and family child care programs where a small number of children receive care from one or more adults in a home setting [2]. The ECE workforce that supports this system is comprised of populations that are considered higher risk for chronic disease. ECE providers tend to earn low wages ($11.17/h versus $18.50/h for all occupations), and more than half of all ECE providers are enrolled in at least one public support program, such as Medicaid or Supplemental Nutrition Assistance. Nearly all ECE providers are female and they are disproportionately women of color [3] (see Table 1 for more demographic characteristics of the ECE workforce in the USA).

**Table 1:** Demographic characteristics of child care workers in the USA, 2014

	Child care workers	All other workers
Gender		
Men	4.4%	53.7%
Women	95.6%	46.3%
Citizenship status		
US born	81.2%	83.5%
Naturalized US citizen	8.3%	7.8%
Non-naturalized immigrant	10.5%	8.7%
Race/ethnicity*		
White	60.1%	66.1%
Black	14.1%	10.6%
Hispanic	19.8%	15.7%
Asian	3.9%	5.8%
Other	2.1%	1.9%
Education		
Less than high school	8.6%	8.0%
High school	30.5%	27.2%
Some college	39.4%	29.7%
Bachelor’s degree	17.7%	22.8%
Advanced degree	3.8%	12.4%
Age		
18–22	15.4%	7.3%
23–49	55.7%	59.1%
50+	29.0%	33.7%

*Race/ethnicity categories are mutually exclusive (i.e., white non-Hispanic, black non-Hispanic, and Hispanic any race)

Adapted with permission from Economic Policy Institute [3]

Just 15% of the ECE workforce receives employer-sponsored health insurance, compared to 49.9% of workers in other occupations [3]. According to the Early Childhood Workforce Index, in 2012, prior to the full implementation of the Affordable Care Act (ACA), nearly 25% of center-based staff, and 21–28% of home-based providers were uninsured. A few studies conducted after Medicaid (publicly funded health insurance) expansion have shown that more ECE providers are enrolling in Medicaid as it is made available in their states [1]. However, given that only 33 states have expanded Medicaid coverage and lack of publicly available data, it is difficult to get the full picture of health insurance coverage for this population [1]. Health disparities and increased chronic disease risk among people with low socioeconomic status, racial minorities and the uninsured is well-documented [4–6]. Despite a potentially elevated risk for chronic disease, little research has been done on the health of the ECE workforce or on the effectiveness of non-clinical health interventions that may reduce chronic disease risk within this population.

Worksite wellness programs are growing in popularity as a way to improve employee productivity, retention, and job satisfaction and lower healthcare costs [7]. These programs typically focus on modifiable health behaviors such as smoking, diet, physical activity, and receiving cardiovascular disease screenings. However, worksite wellness programs are not widely available to the ECE workforce [8, 9]. Most ECE settings operate independently or within small organizations that may not have the resources to offer worksite wellness programs. One exception is ECE settings that are embedded within larger organizations (e.g., large companies, universities, government settings) where the ECE workforce has access to wellness programs offered organization-wide.

The purpose of this project was to establish the current state of the science with regards to ECE workforce health status as well as begin to understand the current state of practice.

## Methods

We used a scoping review approach to collect and describe this emerging field [10, 11]. This approach allows for a more flexible integration of different types of evidence, as is appropriate for a new field. We compiled both published, peer-reviewed evidence, and information on unpublished interventions and programs in an effort to identify what is currently known about the health status of the ECE workforce and examples of interventions currently being implemented to improve health status.

### Data sources

We used two different procedures to gather data for this scoping review, one focused on the published literature and another on practice. For the published literature, we first searched both PubMed and Google Scholar. Search terms encapsulated the environment (child care or daycare or early care and education or preschool or head start), the target population (workforce or employee or worksite or teacher or provider) and at least one component of health (health or wellness or stress or well-being). We also conducted hand searches of the reference sections of relevant papers and forward citation searches.

For practice, we disseminated a call for interventions via several different professional channels focused on the ECE workforce in the USA (e.g., Child Care Aware of America, the CDC’s Division of Nutrition, Physical Activity and Obesity’s Early Care and Education News Blast, Nemours Children’s Health System). In addition, the authors reached out directly to individuals and organizations to inquire about any relevant programs.

### Study selection

Following the searches, a multi-step review process was used to select publications to include in the review. For the published literature, inclusion criteria consisted of both descriptive and intervention studies focusing on the target population (ECE workforce in the USA), published in English within the past 10 years. We did not limit the types of study designs that were included in this review and so we did not assess the quality of the resulting evidence. Given the relatively small size of the evidence base in this area, we included otherwise unpublished dissertations, Master’s theses and organizational reports (e.g., government, non-profit). To assess whether articles met these criteria, titles and abstracts were first reviewed by the first author for relevance and duplicates were deleted. Next, full-text of remaining papers (*n* = 43) were reviewed to confirm that each paper met the inclusion criteria. Lastly, discussion with the whole author team was used if there was ambiguity about a particular paper. During this process, six articles were excluded because they were conducted outside of the USA and two were excluded because they were commentary pieces and did not contain primary data.

For programs, we included any program targeting some component of ECE workforce health that has been offered in the USA at least one time within the past 3 years. We excluded studies and programs that focused on only acute health topics (e.g., infectious disease or injuries). Both published intervention studies and unpublished programs were included.

### Data extraction

For publications, data on target population/sample, methods, and results were gathered from the full-text of the articles. One author (MM) did an initial extraction of each article which was reviewed and confirmed by the lead author to confirm the accuracy of the extraction and summarization. Following extraction, we summarized the resulting evidence qualitatively and present themes across the evidence base. Only quantitative data on health status or behaviors was included (e.g., attitudinal or knowledge data were excluded from extraction).

For practice, organizations and individuals were asked to complete a form providing information about the intervention design (e.g., target behaviors/outcomes; activities or program components) and any evaluation data or results. We followed up with individuals as needed to obtain complete information.

## Results

In total, 26 articles met the inclusion criteria for health status (Table 2). Two articles were excluded from the health status analysis because they involved qualitative methods and did not present prevalence estimates [36, 37]. The themes from these papers were, however, integrated within the results section.

**Table 2:** Summary of research articles describing the health status of the early care and education workforce identified in a scoping review

Source (author, year)	Target population (*n*)	Methods	Health outcome(s) (measurement tool)
Becker et al., 2017 [12]	HS teachers (*n* = 1001) in Pennsylvania	Web-based survey of HS teachers in 37 Pennsylvania HS Programs.	- Depression (CES-D) - Dispositional Mindfulness (CAMS-R) - Workplace stress (JCQ)
Denham et al., 2017 [13]	Female lead teachers from both CCC and HS (*n* = 127) in a Mid Atlantic USA city	Web-based survey of female lead teachers at for-profit, faith-based, and university child care work settings, as well as HS programs.	- Child Care Worker Job Stress (CCW-JSI)
Figueroa and Wiley, 2016 [14]	FCC providers (*n* = 107) in a Midwestern USA city	Web-based or paper survey of licensed FCC providers recruited through local child care resource and referral agencies.	- Physical Activity (Go NAP SACC Physical Activity Self-Assessment)
Grant et al., 2016 [15]	CCC teachers (*n* = 1129) from all 50 states and Washington, DC	Paper survey of teachers from a national sample of ECE programs.	- General Stress (Perceived Stress Scale) - Job-relate emotional exhaustion
Halloran et al., 2018 [16]	HS teachers (*n* = 85) in Rhode Island	Paper survey of HS teachers from 22 HS centers in Rhode Island.	- Fruit and vegetable intake (FVS)
Hibbs-Ship et al., 2015 [17]	HS staff (*n* = 154) in Colorado	Paper surveys of teaching, administrative, program, foodservice, and transportation staff at HS locations. 25 completed additional individual telephone interviews.	- Physical activity - Diet - Barriers to exercise - Self-report of healthy lifestyle
Hindman and Bustamante, 2019 [18]	HS teachers (*n* = 362) in all 50 states and Washington, DC	Paper survey of HS teachers conducted twice within the same year, once in fall and spring.	- Depression (CES-D)
Jennings, 2015 [19]	Teachers from both CCC and HS (*n* = 35) in California	Web-based survey and follow up phone interview of 21 teachers working in privately funded independent preschools and 14 HS teachers.	- Depression (BDI) - Burnout (MBI) - Mindfulness (FFMQ)
Jeon et al., 2018 [20]	Teachers from CCC (*n* = 1129) in all 50 states	Mailed survey of preschool classroom teachers in CCC and public pre-kindergarten programs.	- Depression (CES-D) - Stress (Perceived Stress Scale)
Jeon et al., 2019 [21]	Teachers from CCC (*n* = 207) in a Southern state in the USA	Paper survey of CCC teachers.	- Depression (RAND Health) - Job-related stress (CCW-JSI)
Ling, 2018 [22]	HS teachers (*n* = 80) in Michigan	Web-based survey sent to each HS center supervisor to distribute to all HS teachers.	- Physical Activity (IPAQ) - Depression (RAND Health) - Diet - Quality of Life (SF-36) - Self-reported height and weight
Linnan et al., 2017 [23]	Teachers and administrators from CCC (*n* = 674) in North Carolina	Web-based and paper surveys of 118 administrators and 556 staff from 74 CCCs in North Carolina.	- Health behaviors (CHART) - Dietary Intake (Dietary Screener Questionnaire and the Diet History Questionnaire) - Tobacco and E-Cigarette (BRFSS) - Sleep (PSQI) - Depression (CES-D) - Measured height, weight, waist circumference, heart rate, and blood pressure - Physical activity (1 week of accelerometer monitoring using a GT3X ActiGraph monitor)
Magerko, 2016 [24]	FCC providers (*n* = 165 for survey) in Illinois	Survey of FCC providers.	- Weight status (BRFSS) for BMI - Perceived stress (PSS-14) - Smoking (WHO) - Nutrition (BRFSS) - Physical activity (GPAQ) - Life satisfaction (BRFSS) - Sleep (BRFSS) - Chronic diseases (BRFSS) - Depression (CES-D 20)
Magerko, 2016 [24]	FCC providers (*n* = 67 for measurements) in Illinois	Survey and measurements of FCC providers.	- Measured height and weight - Body composition - Measured blood pressure - Measured blood cholesterol - Dietary intake (24-h recall) - Physical activity (*n* = 28 providers; accelerometer) - Health behaviors (BRFSS)
Ota et al., 2013 [25]	Child care providers from both CCC and FCC homes (*n* = 39) in Utah	Survey of primary and secondary caregivers from 11 family home providers, 18 family group providers, and 10 CCC providers.	- Stress (PSI-SF)
Roberts et al., 2017 [26]	Teachers from CCC (*n* = 1640) in one Midwestern state in the USA	Paper surveys of educators within 1063 schools or centers.	- Work related stress (CCW-JSI) - Depression (CESD-10) - Health Insurance
Sandilos et al., 2015 [27]	Teachers from both CCC and HS (*n* = 59) in Northeastern and Southeastern USA	Paper survey of teachers from HS and preschool centers serving children who receive free or reduced lunch.	- Depression/Emotional Health (Kessler Psychological Distress Scale) - Stress (Job Control portion of CCW-JSI)
Sharma et al., 2013 [28]	HS Teachers and teachers’ aides (*n* = 213) in Texas	Paper survey of HS teaching staff (i.e., teachers and teachers’ aides).	- Dietary behavior (semi-quantitative food recall questionnaire) - Self-report height and weight
Snyder and Hill, 2018 [29]	HS teachers and other staff (*n* = 312) in Ohio	Web-based survey of teaching, family support, health, administrative, and support staff of the HS agency.	- Stress (PSS-4) - Mindfulness (MAAS) - Mental Health (NHANES and BRFSS) - Chronic conditions (NHANES) - Smoking (BRFSS) - General Health Status (BRFSS)
Song et al., 2016 [30]	HS teachers and other staff who were aged 18 years or older (*n* = 307) in Michigan	Web-based survey of teachers and other staff from 17 Michigan Migrant and Seasonal HS centers.	- Self-reported height and weight - Food security (US household food security survey module)
Swindle et al., 2018 [31]	Early childhood educators (*n* = 307) in Arkansas	Survey completed on location or at a conference for ECE providers.	- Dietary intake (FMI) - Food insecurity (Household Food Security Survey)
Tovar et al., 2017 [32]	FCC providers (*n* = 166) in North Carolina	Paper surveys completed by providers at on-site visits.	- Physical activity (BRFSS) - Dietary intake (Brief Block Food Frequency Questionnaire) - Sleep (Medical Outcomes Study sleep scale) - Stress (Perceived Stress Scale) - Measured height and weight
Ward et al., 2018 [9]	CCC directors and participating staff (*n* = 553) in North Carolina	A combination of an objective physical activity measure, physical measurements, web-based and paper-based surveys, and an environmental assessment.	- Health behaviors (CHART) - Smoking (BRFSS) - Physical activity (7 days of accelerometer wear) - Dietary intake (Dietary Screener Questionnaire and the Diet History Questionnaire) - Sleep (Society for Behavioral Medicine’s Common Data Elements and Pittsburgh Sleep Quality Index) - Depression (CESD)
Whitaker et al., 2013 [33]	Female HS staff (*n* = 2122) in Pennsylvania	Web-based survey of female HS program directors, managers, classroom teachers, home-based visitors, and family service workers. Staff from 66 Pennsylvania HS programs.	- Depression (CES-D) - Chronic disease diagnoses (BRFSS, NHIS) - Health-related quality of life (NHIS and BRFSS)
Whitaker et al., 2015 [34]	HS Teachers and assistant teachers (*n* = 1001) in Pennsylvania	Web-based survey of teachers and assistant teachers from 37 HS programs in Pennsylvania.	- Depression (CES-D) - Financial well-being
Witherell, 2013 [35]	CCC employees (*n* = 101) in Michigan	Paper survey of CCC head or lead teachers, group leaders, assistant teachers, and aides.	- Depression (CES-D; BSI)

*FCC* family child care, *CCC* child care centers, *HS* Head Start

### Physical health

Thirteen studies described some component of physical health including diet quality (11 studies), physical activity (8 studies), and height/weight/BMI (7 studies). Five studies each measured sleep and smoking and four studies measured chronic disease status.

#### Diet quality

Ten studies measured fruit and/or vegetable consumption. The proportion of providers meeting recommendations for fruit and vegetable consumption (3.5 cups or 5 servings per day) varied from 22.5% of Head Start staff [17] to 50% of family child care (FCC) providers [32]. Mean fruit and vegetable consumption exceeded recommendations in one study of Head Start teachers [16] but were below recommendations in one study of child care center (CCC) directors and staff [9].

#### Physical activity

Five studies explored the proportion of providers meeting national physical activity requirements (e.g., 150 min per week of moderate to vigorous physical activity); the results included 27% of CCC employees [23], 29.4% of CCC directors and staff [9], and 55% of Head Start teachers [22] met these recommendations. Approximately 40–50% of FCC providers in two studies reported meeting guidelines [24, 32]. Providers across four studies reported large quantities of sedentary time; one study of Head Start (HS) teachers found they spent 291.69 min (4.9 h) per weekday sitting [22]; two studies of CCC employees found a mean of 481 and 513.6 min (8–8.6 h) of sedentary time per day, respectively [9, 23]. One study of FCC providers found that nearly one third (32.8%) reported nine or more hours of sedentary time per day [24].

#### Weight status

Seven studies assessed height and weight and converted to BMI; high levels of overweight and obesity (defined as BMI > 25) were reported. Rates of overweight and obesity included between 73.5 and 80.1% for HS staff [22, 28, 30], 71% and 89.9% for FCC providers, and 88.5% and 87.2% of CCC staff [9, 23]. Nationally, 71.6% of adults are overweight or obese [38].

#### Sleep

Three studies explored whether FCC providers were meeting sleep recommendations (7 or more hours of sleep per night); between 43.4 and 56.7% were regularly meeting these goals [24, 32].

#### Smoking

A small proportion of providers reported being current smokers; from 15.6% of CCC staff [23] to just 7.5% of FCC providers [24].

#### Chronic disease status

Three studies assessed diabetes prevalence and found similar rates (10.6% for FCC, 10.4% for FCC, 11.9% for HS) [24, 33] compared to just 7.8% of a comparable national sample [33]. Rates of diagnosed high blood pressure included 22.3% of HS staff and 36% of FCC providers [24, 33]. One study of HS staff found higher rates of four additional chronic diseases and conditions (severe headache/migraine, lower back pain, obesity and asthma) as compared to a similar national sample [33].

### Mental health

In total, we found 21 studies that included measurement of mental health including depression (15 studies), stress (8 studies), and mindfulness (3 studies).

#### Depression

Fifteen studies explored depression levels of ECE providers; from two samples of FCC, approximately 23% reported a depressive disorder or diagnosis [24]. Among the five analyses of HS staff, one study found an average Center for Epidemiologic Studies—Depression (Scale) (CES-D) of 10.8 (at or above 16 is considered screening positive for depression) [12]; another found 35% of respondents with at least moderate depression at two time points during the year [18]; and Ling found that 31% of HS teachers were experiencing depressive symptoms [22]. In two analyses from the Pennsylvania Head Start Survey [33, 34], approximately 24% of respondents had a CES-D score at or above 16. This contrasts with only 17.6% of a national comparison sample with similar demographics. The eight studies that included CCC staff generally found lower rates of depressive symptoms; fewer than 19% of respondents in one study of CCC staff scored above 16 on the CES-D [35] and only 8.9% of respondents in another study had clinically significant depression [26]. Two analyses of data from CCC providers in North Carolina found higher rates of depression (34.9–36% with a CES-D at or above 16), compared to the national average of 12.3% of women ages 40–59 [9, 23].

#### Stress

While only one of the eight studies that explored stress levels of providers included prevalence data on high stress, several explored the impact that stress levels have on performance. One study of 39 providers (CCC staff and FCC providers) found that higher levels of provider stress were associated with *lower* child engagement in the classroom [25]. Another study found that stress levels were associated with a greater intention for teachers to leave rather than stay in their positions [15]. In a survey of FCC providers conducted by Tovar and colleagues (2017), 62% of respondents had a high stress score on the Perceived Stress Scale [32].

#### Mindfulness

Three studies measured mindfulness traits among ECE providers, each with a different measure (CAMS-R, FFMQ, MAAS), which makes comparisons across samples difficult.

### Interventions

Following the literature and practice searches, seven published and four unpublished programs were identified. Details on the audience, target behaviors/outcomes, activities/components, and evaluation results (if available) are included in Table 3.

**Table 3:** Summary of wellness programs targeting the early care and education workforce identified in a scoping review

Source	Target population	Target behavior(s)/outcomes	Activities/components	Evaluation results (if available)
Published literature				
Esquivel et al., Children’s Healthy Living Program for Remote Underserved Minority Populations in the Pacific Region [39]	HS teachers in Hawaii	Teacher health status and health behaviors; knowledge, misconceptions, beliefs and priorities on nutrition and childhood obesity preventions	Staff wellness classes as part of a larger, 7- month long intervention targeting policy and classroom-level changes. Monthly classes focused on benefits of physical activity (PA), stretching and PA ideas at work; benefits of healthy eating and food tasting, and; stress management.	Impact on HS teacher health not provided
Messiah et al., Healthy Caregivers - Healthy Children (HC2) [40]	CCC in Florida		Using a train the trainer approach, policy changes regarding nutrition, physical activity and screen time were pursued with participating CCCs. Teachers and parents also received six monthly workshops to support their role as healthy role models for children.	Not available
Arandia et al., Caring and Reaching for Health’s Healthy LIfestyles Intervention (CARE) [8]	CCC in North Carolina	Nutrition and physical activity	Multi-component, theory-based intervention includes a kick-off event and educational workshop and three 8-week campaigns (6 months total duration). Each campaign focused on a different PA-related topic and included print materials, goal setting and self-monitoring, tailored feedback, email and text prompts and coaching for CCC directors.	Pilot results suggested that the intervention resulted in significant decreases in provider BMI and smoking along with increases in physical activity and fruit/vegetable intake
Gosliner et al. [41]	CCC in California. 98% of participants were female. 91% were aged 25 to 64 years. 49% were white, 24% Asian or Pacific Islander, 17% African American, 13% Hispanic/Latino, and 6% other. 52% of participants had attended some College education, 21% had received an associate degree or 16% had a bachelor’s degree.	Physical activity (measured via accelerometry); secondary outcomes include other health behaviors (e.g., diet, weight, smoking), physical health indicators (e.g., BMI, blood pressure, fitness tests)	Wellness program added to existing intervention focused on obesity-related policy change in CCCs. Activities offered over 9 months included day-long kick-off training, monthly newsletters and paycheck insert, and a staff walking program.	Compared to a control group, intervention participants reported significantly lower sugar sweetened beverage consumption; significantly higher ease of engaging parents in discussion of child’s eating and comfort talking to parents about child’s PA. Other differences were not significant.
Herman et al., Eat Healthy, Stay Active! [42]	HS teachers in Pennsylvania, Texas, Arizona, Rhode Island and New York. 96% of participants were female. 56% were white, 1.4% Asian or Pacific Islander, 14.3% African American, 17.5% Hispanic/Latino, 3.6% Native American, and 2.4% other. 15% had a high school diploma or GED, 17% had an associate’s degree, and 65% had a bachelor’s degree. 60.5% of participants were married, 91% worked part-time, and 4% worked full-time	Nutrition and physical activity; weight change, knowledge and behavior	Six-month multi-component intervention targeting nutrition and PA among children, teachers and parents. Staff received 1 day of training on intervention components and then delivered workshops to parents and children.	Significant decrease in staff BMI, significant increase in knowledge, diet and physical activity.
Jones, Teacher be well: Mindfulness based stress reduction [43]	CCC on military base in San Diego, California	Mindfulness, workplace stress	Two-hour mindfulness workshop that included lecture, discussion, exercises coupled with take-home resources. Printed materials supporting the content were available in the break room.	No significant impact on mindfulness or workplace stress among participants (*n* = 27). Process evaluation showed that the program was feasible and participants were satisfied with the program.
Ostbye et al., Keys to Healthy Child Care [44]	FCC providers in North Carolina. 57.5% of providers were African American, 40% were White, and 2.5% were Asian. Mean age of providers was 46.2 years.	Physical activity, diet quality, height and weight; and FCC environment	Nine-month, three component intervention guided by Social Ecological Model and Social Cognitive Theory. Three months each are spent on: Healthy You, Healthy Home and Healthy Business concepts. A health behavior coach is paired to each participant and provides one group workshop, one in-person visit to the FCC home, three tailored phone calls and a written toolkit.	No results available
Unpublished				
Be Well, Care Well (Medical University of South Carolina, Boeing Center for Children’s Wellness)	CCC providers in targeted regions of South Carolina	Overall wellness, resilience, physical activity, healthy diet, stress, job satisfaction	Well-Being Coaches work with center-level committees composed of three or more members (one administrator, one or more teachers, one or more parents) to identify well-being goals. Through weekly, on-site visits the coaches work with committee to achieve selected well-being goals, provide support and incentives and connections to local resources.	Not available.
Create Healthy Futures (Penn State Extension Better Kid Care in collaboration with UTHealth School of Public Health) [45]	ECE providers across multiple settings in Cleveland, Ohio. 97.3% of participants were female. 41.5% were African American. Mean age was 43.5 years	Improve consumption of healthy foods, raise awareness of the nutrition environment, support ECE professionals as role models for children and families	Four-hour, self-paced, online program developed using Social Cognitive Theory and the Social Ecological Model. Content includes videos, reflection activities, downloadable handouts and action planning. Topics covered include basic nutrition information, healthy eating strategies, food environment and food culture reform. Participants also receive 6 weeks of peer coaching sessions to support healthy behavior change.	One pilot study conducted with 111 ECE professionals from four facilities. Participants showed increase in nutrition knowledge, decrease in perceived barriers to promote healthy nutrition in the classroom, and improved wellness support at their workplace. Process evaluation results found the program to be helpful, acceptable and feasible. Additional study on the effect of the program is underway with a larger sample of Head Start professionals.
Building Well-Being. Resilient Nourished Active. (Spokane Washington Regional Health District)	Early learning staff (including cooks) and directors in Spokane, Washington	Social emotional well-being and self-regulation; healthy nutrition and menus; promoting active play among children	Year-long program focused on emotional and physical well-being of both staff and children. One-on-one coaching, monthly online webinars, teaching tools provided to participants and in-person interactive trainings on healthy eating and active play for staff with an emphasis on how nutrition and physical activity influence mood, behavior and health of staff.	Pilot underway with six centers (125 teachers). Evaluation will measure impact on child- and adult-level outcomes including proportion modeling deep breathing techniques when addressing conflict and teacher engagement in physical activity with children.
YMCA child care facilities and camp (Greater Wichita Kansas YMCA)	Staff of YMCA child care facilities and camps in Greater Wichita, Kansas	General wellness, healthy eating, physical activity and mind-body balance	Staff receive free YMCA memberships, annual biometric screenings, voucher for preventive health visits, staff games, and quarterly health challenges. Staff also have access to monthly webinars focusing on wellness topics.	Not available.

#### Audience

The majority of the programs were delivered to center-based teachers and staff, with only one developed specifically for family child care providers [44, 46]. Others focused on Head Start staff [39, 42] or a broader audience of providers including FCC (33, Building Well-Being Resilient).

#### Target behaviors and program components

All but one program included nutrition/healthy eating components; most included physical activity and five included stress or other mental health-related targets. The majority of the published interventions were larger, multi-component interventions that included some staff wellness component (as opposed to a stand-alone staff intervention). Most were also conducted over a long period of time ranging from 6 to 12 months with multiple pedagogical techniques (e.g., workshops, print materials, individual or group coaching). Only one identified program (YMCA child care) described ongoing efforts that are always available to employees. All others represented one-time interventions, with the majority sponsored by outside organizations and/or researchers working in conjunction with providers.

#### Evaluation result

Limited evidence on the effectiveness of these interventions is available. Among the published interventions, only four included participant-level impact data. The CARE Intervention has shown preliminary evidence of impact; a pilot study showed positive impact on BMI, physical activity, fruit and vegetable intake, and smoking [8]. Another intervention, offered over 9 months as part of a larger intervention targeting center-based providers in California, showed positive impact on only sugar sweetened beverage consumption [41]. The Eat Healthy, Stay Active! program has been shown to positively impact provider BMI, diet, physical activity, and health-related knowledge [42]. A one-time mindfulness workshop did not show significant impact on provider mindfulness or workplace stress [43]. Among the unpublished programs, only one has evaluation results available. The Create Healthy Futures program, a 4-hour online program, showed improvements in nutrition knowledge and perceived barriers to promoting wellness in the classroom [45].

## Discussion

This scoping review provides a well-rounded picture of the health status of the ECE workforce in the USA along with description of interventions recently implemented to improve health in this population. From the health status literature, it is clear that many ECE providers struggle with chronic disease risk behaviors (e.g., healthy eating, sedentary time) and mental health challenges (e.g., stress and depression). These challenges persisted across setting (e.g., HS versus CCC; teachers versus directors). This is likely exacerbated by the low socioeconomic status among a majority of ECE providers, as well as lack of access to health insurance through their employers [1]. Given that many child care centers and homes cannot afford to provide health insurance, introducing workforce wellness programs may be an affordable work-around to addressing chronic disease risk behaviors and mental health challenges. Specific recommendations on health behavior targets likely vary depending on the ECE type (e.g., FCC versus HS) and the local context; however, it is clear that there is room for improvement across the board for physical activity and diet. Many providers in the included studies were overweight or obese, raising their chronic disease risk.

For physical activity, three studies found that ECE providers spend between 4 and 8.6 h/day in sedentary activity levels, which have been shown to be associated with obesity and certain cancers, independent of physical activity levels [47, 48]. Thus, interventions targeting sedentary time may be especially warranted in this population.

Insight can also be drawn from research done on ECE providers in other countries, as well, as workplace stress is a common factor for child care providers in many countries. For example, Corr et al. looked into the relationship between Australian family child care educators’ mental health and working conditions, finding that social support was associated with higher mental well-being [49]. Similarly, Nislin et al. found that teamwork was critical to supporting the well-being of ECE providers in Finland [50]. Both of these studies were conducted in countries where there is significantly more government financial support for ECE providers (in Australia, for example, over 70% of ECE providers said their income was “enough” or “more than enough” to meet needs) [49], as well as nearly universal health care access, which eliminates some of the socioeconomic stresses that American ECE providers face.

The interventions and programs included in our review provide a wide range of options for future efforts. Many were embedded in larger, facility-wide programs targeting obesity prevention in adults and children. Research shows that Whole Systems Approaches are most likely to have a significant impact on complex public health challenges including obesity [51]. Most were conducted over 6 months or more, illustrating that impact on chronic disease and mental health outcomes requires a significant investment of time and support. Given the diversity within the ECE workforce, it is also likely that different intervention approaches will need to be used for different audiences. For example, interventions originally developed for center-based staff may not be appropriate for family child care providers who have different access to colleagues (e.g., social support) and administrative supports to facilitate wellness programming.

There have been calls in the literature for an increase in practice-based evidence (PBE) or studies that combine the rigor of the scientific process with the realities of implementation in the real world [52]. Researchers interested in this approach can use the list of interventions provided here as a jumping off point to develop collaborative research projects to assess the implementation and impact of these interventions on the ECE workforce. Additional research should be done to determine the extent to which these programs align with best practices in worksite health promotion developed by the CDC [53].

### Limitations

Many of the descriptive studies did not include a comparison group of individuals with similar demographics but different occupations. Such a group would improve understanding of whether the health status of the ECE workforce is different from others with similar demographic characteristics, especially age, education level, and income. Our programs only included one facility-level wellness intervention (YMCA child care), though ECE professionals connected to larger employers, such as corporations or universities, are likely have access to employee wellness programs. A recent national survey found that nearly 50% of workplaces in the USA with at least 10 employees offer some sort of employee wellness programming [54]. While none of those programs responded to our call for programs, the authors are aware that these initiatives exist across the country despite their relative absence from our review.

## Conclusions

The results of this scoping review suggest that additional research into the health status of the ECE workforce is needed to properly categorize chronic disease risk across the diversity of the workforce. The limited body of evidence available for review paints a picture of a workforce in need of health promotion intervention to address high levels of mental and physical health challenges, some above and beyond peers with comparable demographic characteristics. Several promising interventions were identified from both the published and unpublished literature; these interventions should be further implemented and evaluated to assess their impact on the workforce.

## Data Availability

Data sharing is not applicable to this article as no datasets were generated or analyzed during the current study.

## Abbreviations

BMI: Body mass index
CCC: Child care center
CDC: Centers for Disease Control and Prevention
CES-D: Center for Epidemiologic Studies—Depression (Scale)
ECE: Early care and education
FCC: Family child care
HS: Head Start
PBE: Practice-based evidence
YMCA: Young Men’s Christian Association
